# Diffracting the rays of technoscience: a situated critique of representation

**DOI:** 10.1007/s10202-011-0099-5

**Published:** 2011-11-23

**Authors:** Federica Timeto

**Affiliations:** 1 University of Plymouth, Planetary Collegium, UK; 2 M-Node at Nuova Accademia di Belle Arti (NABA), Milan, Italy; 3 University of Urbino Carlo Bo, Urbino, Italy

## Abstract

This essay focuses on the possibility of adopting a representational approach for technoscience, in which representation is considered as a situated process of dynamic “intra-action” (Barad 2007). Re-elaborating the recent critiques of representationalism (Thrift 2008), my analysis begins by analysing Hayles’s situated model of representation from an early essay where she explains her definition of constrained constructivism (Hayles [1991] 1997). The essay then discusses the notions of figuration and diffraction and the way they are employed by Haraway in many of her writings for her critique of technoscience (Haraway 1991, 1997). Finally, after considering diffraction through Barad’s reading of this practice in the context of her theory of agential realism (2007), it shows the links that relate constrained constructivism, situated knowledge and agential realism, and the way all of them work at “diffract[ing] the rays of technoscience” (Haraway 1997: 16) through an alternative representational practice.

## Résumé

Cette rédaction de focalise sur la possibilité d’adopter une approche représentationnelle envers la technoscience, dans laquelle la représentation est considérée comme un processus située d’ “intra-action” dynamique (Barad, 2007). En ré-élaborant les critiques récentes de représentationalisme (Thrift, 2008) mon analyse commence par l’analyse du modéle situé de représentation de Hayle, tiré d’un essai antérieur dans lequel elle explique sa définition du constuctivisme contraint (Hayles [1991] 1997). Puis, je traite les notions de figuration et de diffraction et la manière dans laquelle elles sont employées par Haraway dans beaucoup de ses publications concernant sa critique des technosciences (Haraway 1991, 1997). Enfin, après avoir considéré la diffraction à travers les écrits de Barad sur cette pratique dans le contexte de sa théorie de réalisme agentiel (2007), un lien devient évident entre le constructivisme contraint, la connaissance située et le réalisme agentiel, ainsi que la manière dans laquelle ils participent dans la “diffraction des rayons de la technoscience” (Haraway 1997: 16) par une pratique alternative représentationelle.

## Abstract (de)

Der folgende Essay befasst sich mit der Möglichkeit, in der Analyse von Technowissenschaft einen repräsentationalen Ansatz zu verfolgen, der Repräsentation als situierten Prozess dynamischer Intra-Aktion (Barad 2007) versteht. Mit Bezug auf rezente Kritik des Repräsentationalismus (Thrift 2008) setzt meine Analyse bei Hayles’ ([1991] 1997) situiertem Repräsentations-Modell und dem von ihr propagierten *constrained constructivism* an. Der Essay diskutiert daraufhin die Konzepte der Figuration und Diffraktion in Haraways (1991, 1997) Analyse von Technowissenschaft sowie Barads Analyse von Diffraktion im Kontext ihres *agential realism* (2007). Schließlich werden Gemeinsamkeiten von *constrained constructivism*, situiertem Wissen und *agential realism* dargestellt; insbesondere deren Versuch, durch alternative repräsentationale Praktiken “die Strahlen der Technowissenschaft zu beugen” (Haraway 1997: 16).

## Introduction

According to Haraway, three “crucial boundary breakdowns” have put an end to the “border war” of Western science and politics today, which involve the territories of production, reproduction and imagination (Haraway 1991: 151–153); these boundaries are those between human and animal, organism and machine and the physical and non-physical realms. Hence, Whatmore (2006) lists some important shifts in scholarship that reflect such breakdowns, involving many theoretical fields, from cultural geography to science and technology studies. The first shift that Whatmore identifies is the relocation of agency in practice and performance, and a re-embodiment of theory itself, which marks the passage from discourse to practice. The second is the shift from meaning to affect, involving a rediscovery of the precognitive and of its role in sense making as a “force of intensive relationality” (ibid.: 604). The third, a consequence of the previous dislocation, is the shift from the human to the more-than-human, or from society conceived as a closed and exclusively human whole to a multiplicity of assemblages constituting a heterogeneous sociomaterial fabric. Finally, the fourth shift is the move from a politics of identity to a politics of knowledge, the way this is produced, negotiated or contested according to different sociotechnical contexts and distributed practices (ibid.: 603–604). According to a similar approach, knowledge does not stand outside the world it represents, but emerges from it and is enmeshed in it, being in this sense *situated*; given that representations are social facts, we cannot get rid of them: it doesn’t matter if they are true or false; what matters is, rather, how they work, and why (Rabinow 1996: 28 ff.).

In her analysis, Whatmore directly quotes Barad to reinforce her argument that matter does matter, and that it also “comes to matter,” performatively and processually (Whatmore 2006: 605); Whatmore also refers to Barad in her previous work in which, discussing the importance of distributed agency and the material-semiotic practices of the constitution of the subject, she draws on Barad’s notion of “intra-action” (Whatmore 2002: 4, 57), which the latter formulates in the context of her philosophy of “agential realism” (see below). In what follows, a compared analysis of Hayles’s theorization of constrained constructivism, Haraway’s concept of diffraction and Barad’s agential realism aims to reconceptualize the role of representation for technoscience as an intra-active practice embedded and embodied in hybrid sociotechnical networks. If “representationalism takes the notion of separation as foundational” (Barad 2007: 137), talking of representation as intra-action means considering the “*mutual constitution of entangled agencies*” (ibid.: 33) which do not precede, but rather emerge through their intra-acting processes.

Whereas conventional epistemologies have conceptualized science as a “set of representations of reality,” interactionist (or, rather, intra-actionist) approaches consider science as intrinsically technological and performed through different practices, interpretations and applications (Harding 2008: 186–187).^1^ Scientific knowledge cannot accurately represent the world from a distance, let alone its objectivity, but only show**s** how the world effectively works and how representation can adequately fit such workings (Latour 1987; Haraway 1997). Let us think, for instance, of the “less false accounts” or “less false beliefs” about the world in the sense that Harding intends them in her theory of standpoint epistemology, “ones, apparently, as far as we can tell, less false than all *and only those* against which they have so far been tested” (Harding [1997] 2004: 256). These are provisional truths whose standards vary over time and space, but which are nonetheless useful, effective notions against both universalist and relativist claims. They are *adequateinterventions* that replace the search for a semantic match between sign and things with the search for efficacy (Harding 2003: 156–157).

## Beyond representationalism

In the last two decades, the debate around the issue of representation has occupied several different fields, primarily as a reverberation of the anti-realist constructivist turn that has permeated postmodern philosophical debate.^2^ Discussing the different traditions of the conceptualization of representation as the knowledge of reality, Peschl and Riegler (1999) show the change of focus that has occurred in the last decades, from an attempt to grasp the structure of the environment and map it onto a representational structure, according to an analogical correspondence between signs and things, to an awareness of representation as a dynamic and generative process where environment, rather than reality, only constrains representation instead of determining its outcomes.

According to a radical realist position, the domain of our experiences as *Wirklichkeit* equates the world of things as *Realität*. Classical representational theory transforms *Wirklichkeit* into a function of *Realität*. Only in a dialectic materialistic perspective representation is re-contextualized and considered as the result of an interaction between the observer, the observed object and the context where observation takes place. But if we go further and adopt a self-referential framework, drawing on the theory of autopoietic systems, we can definitely drop the search for an external reality (without needing to either deny or affirm its ontic existence): in this case, representation is described as the perception of relations among the element of the observed and self-observing system, which is characterized by its operational closure. Once we consider representations not as passive, however, accurate, reflections of an independent reality, but as active constructions and viable, embodied and contingent processes of knowing, we can continue to employ them and at the same time disengage them from a correspondence with reality (and representationalism in a realist sense).

The acknowledgement of the agency of matter and of the hybrid connections between theory and practice, human and non-human beings, takes the form of a strong critique of representation in non-representational theory in particular. This, in most cases, associates representation with the metaphysics of visualism, although, to paraphrase Pickering (1994), when vision is delinked from “the representational idiom” and rather aligned with the “performative idiom,” a recovery and redefinition of visuality always appears possible. The terms of the debate regarding non-representational theory were initially assessed in the field of human geography, but soon turned out to be of interest for many other theoretical domains, such as feminist studies, performance studies and science and technology studies (cf. Lorimer 2005).

In non-representational theory, knowledge is firmly located in matter or, to partially paraphrase the subtitle of Barad’s book (2008), in “the entanglements of matter and meaning;” it is also relationally generated, and by no way solely rational, nor a subjective or even a human property, all assumptions that, on the contrary, belong to the tradition of Western Modernity (Thrift 2008: 122). As Thrift (2008) shows, non-representational theory has its roots in different philosophical traditions and their reciprocal points of contact: for example, feminist theory of performance and feminist spatial analysis, ranging from Butler to Irigaray, the theory of practices drawing on the work of such authors as Bourdieu and De Certeau, and what goes under the name of “biological philosophy,” from Deleuze to the current speculations of biosciences (cf. Thrift 2008: 113; Whatmore 2002). Thrift (2008: 5 ff.) characterizes non-representational theory as the conjoined insistence on a number of aspects. It features a radical empiricism—which is anti-essentialist in character and which also distances itself from constructivism—while aligning itself with the philosophies of becoming, without completely abandoning the lived immediacy of the phenomenological and the precognitive. It includes an anti-subjectivism that disengages perception from the human perceiver and attributes it to encounters among heterogenous forms, or what he calls “new matterings” (ibid.: 22). It relies on practices as being generative of actions rather than being their consequences, thus showing an interest in the “effectivity” of the world (ibid.: 113). It insists on the transhuman co-implication of bodies and things in a network of functions, where embodiment becomes a diffuse situation of shared relationality. It requires an experimental attitude, which owes much to the performing arts and is based on the unpredictability and radical possibility of the evenmental (ibid.: 114). It takes an affective stance that allows the retention of a sort of “minimal humanism” (ibid.: 13) while at the same time being anti-humanistic in a traditional sense, and which translates into an affirmative ethics of responsibility and care. Finally, it has a situational character where space is itself becoming, distributed and networked.

Needless to say, most of these elements can already be found in the theory of situated knowledge, but then this should come as no surprise, given the common root of non-representational theory and Harawaian philosophy in actor-network theory (cf. Latour 2005). Haraway’s politics of representation, however, insists on the importance of vision and images and, recognizing their contemporary pervasiveness, tries to articulate a different, opaque and non-innocent representational attitude which is partial, embodied and situated at the multiple crossings of the material-semiotic field. Her project of situated knowledge recognizes the impossibility of doing without representations; a recovering of the sense of vision, or better, of re-vision, is of the utmost importance for the feminist project of a multidimensional cartography, which is itself a representation of a different kind, being always generated from somewhere, from below and from within the networks of technobiopower. That is why Haraway insists that we pose the following questions:

How to see? Where to see from? What limits to vision? What to see for? Whom to see with? Who gets to have more than one point of view? Who gets blinkered? Who wears blinkers? Who interprets the visual field? What other sensory powers do we wish to cultivate besides vision? (Haraway 1991: 194)

In a sense, a simple opposition to representation advanced in the name of the world of matter is still risky, implicated in the double bind that sees matter and meaning, or the semiotic and the material, as standing in a relation of mutual exclusion. Analogously, says Haraway, if we counterpose situatedness to universalism in a scheme which is still oppositional, we give the false illusion of a symmetry between the two, where each position is seen as purely alternative or reciprocally exclusive (ibid.). Instead, “a map of tensions and resonances between the fixed ends of a charged dichotomy better represents the potent politics and epistemologies of embodied, therefore accountable, objectivity” (ibid.). As Jacobs and Nash (2003) affirm, commenting on recent scholarship in cultural geography, there is no need to dismiss representation altogether, particularly if we consider the importance of a critique and a politics of representation for feminist work, and even if we share the assumptions of non-representational theory. As they put it, we “might insist on attending to the place of image,” so as to keep open a “wider semiotic framework” where words and things interrelate, without contradicting the semiotics of materiality of non-representational theory (ibid.: 273).

## Consistent representations

It is in this direction that Hayles ([1991] 1997) has looked for an escape from the alternative between realism and anti-realism through her notion of “constrained constructivism,” which does not tell us what reality is, but rather what fields of possibility make certain representations “consistent” with reality, and thus practicable for us. As a matter of fact, constrained constructivism is built on an “interactive, dynamic, locally situated model of representation.” Here, the notion of “consistency” replaces that of “congruence.” Whereas congruence implies a one-to-one correspondence between signs and things, based on Euclidean geometry, consistency eschews this oppositional logic; rather than being kept in between the true/false dichotomy, it stands in between the not-true/not-false relation, which is one that subverts the symmetry between affirmation and negation.

What we call “observables,” writes Hayles, always depends on locally situated perspectives according to which different pieces of information about the environment are processed, as demonstrated in the example of the frog’s visuality, which Hayles gives at the beginning of her essay, drawing on the well-known article of Lettvin et al. (1959). For the frog, the Newtonian first law of motion, which for humans applies to every object upon which a force is exerted, does not work equally. A frog’s brain is only stimulated by small objects in rapid movement, allowing it to detect potential prey, whereas bigger or static objects elicit a completely different response. Recognizing, however, that every reality is relative to the observer does not lead Hayles to conclude that systems close in on themselves leaving the world outside, or that perceptions can do without representations at all, as Lettvin, Maturana, McCulloch and Pitts seemed to presuppose, and which Maturana and Varela further developed (Maturana and Varela 1980).

As Hayles notes (1995), even if we agree with the non-representational aspect of perception, we do not necessarily need to believe that “it has no connection with the external world,” particularly when we consider that a relation can also be transformative, rather than solely reflexive (ibid.: 75). And further, she argues *contra* Maturana and Varela, the observer is caught in continuous feedback loops within the autopoietic processes of the system, rendering “the domain of the observer” a convenient fiction (ibid.: 78). Not willing to renounce a term like representation, but rather intending to formulate it differently, as “a dynamic process rather than a static mirroring” (Hayles [1991] 1997), Hayles opts for the way Niklas Luhmann, whose systems are as closed as Maturana’s, nonetheless contemplates much more activity in systems, showing their contingency rather than their inevitability, and thus finds a way to escape the realist/constructivist debate (Hayles 1995: 98). Actually, claims Hayles, “unlike Maturana,” Luhmann

twists the closed circle of tautological repetition (“we do not see what we do not see”) into an asymmetric figure (“one does not perceive when one perceives”). The energy generated by these contradictory propositions rebounds like a loaded spring toward the very term that Maturana’s closure was designed to erase, namely “reality.” What is enacted rhetorically within the structure of this sentence is formalized in Luhmann’s theory by investing the observer with the agency to draw a distinction. By making a distinction, the observer reduces the unfathomable complexity of undifferentiated reality into something she can understand (ibid.: 97).

What Hayles appreciates in Luhmann’s position is that he recognizes “that closure too has an outside it cannot see” (ibid.: 98). This leads us to acknowledge, on the one hand, the fact that “the very interlocking assumptions used to achieve closure are themselves the result of historical contingencies and embedded contextualities.” (ibid.: 98). On the other, it allows for a preservation of the “correlation” or “interactivity” that connections, rather than absolute distinctions, make possible (Hayles et al. 1995: 16). Representations, in this context, appear not as a mirroring of “external” reality, but as “species-specific, culturally determined and context-dependent” processes of dynamic interaction.

In Hayles’s terms (Hayles [1991] 1997), a representation can be consistent with reality, or inconsistent with reality. In the latter case, this suggests that an inconsistent representation does not offer an adequate account of our interaction with what Hayles calls “the flux.” She uses the terms “cusp” and “flux” in order to reformulate the notion of representation and its viability^3^:

On one side of the cusp is the flux, inherently unknowable and unreachable by any sentient being. On the other side are the constructed concepts that for us comprise the world. Thinking only about the outside of the cusp leads to the impression that we can access reality directly and formulate its workings through abstract laws that are universally true. Thinking only about the inside leads to solipsism and radical subjectivism. The hardest thing in the world is to ride the cusp, to keep in the foreground of consciousness both the active transformations through which we experience the world and the flux that interacts with and helps to shape those transformations (ibid.).

Representations, then, connect the sides of the cusp and allow us to ride it. The more representations are consistent, manifesting “local interactions rather than positive correspondences” with the flux, the more their “instrumental efficacy” allows us to “ride the cusp,” so to speak (ibid.). Representations are ruled by constraints, which do not tell us what reality “in its positivity” is, but can tell us when representations are consistent with reality, enacting some possibilities and enabling certain distinctions instead of others. Constraints, then, operate in the making of selections between those representations which are viable and those which are not (ibid.).

**Fig. 1:**
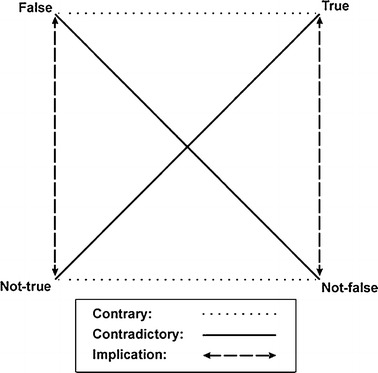
Hayles’s modified Greimas Square

To better show the role of constraints for representations in her theory of constrained constructivism, Hayles adopts and modifies the Greimas Square (Fig. 1).

False and True occupy the top line of the square, so that they are mutually exclusive, since they stand in an exclusionary relation of opposition. Instead, the bottom line is occupied by the couple Not-true and Not-false, whose relation is not an oppositional one: actually, not-false are those representations which are *consistent* with the flux, while not-true are all the *unknown* representations, that is, the not yet practiced representations. This puts not-true and not-false in a relation that is one of consistency and of unknowability, rather than of antithesis—a relation that “folds together the ability to negate with the ability to specify,” that is a relation of denial (the unknown) and assertion (the consistent) rather than of negation and affirmation (ibid.). If I, for instance, look at the pen that lies at my desk, I can surely say that it is an orange pen. However, my assertion is based on the observation of the colour that the plastic case of my pen appears to be. But if someone asks whether I have a black pen to lend, I can surely give them the same pen, given that it writes in black ink, thus is a black pen too. While asserting that my pen writes in black ink, I am not negating the orangeness of my pen, so to speak, but only further specifying something about the way it works.

The difference here is that denial and assertion are what Hayles calls “marked,” or modal, terms, which cannot be assimilated to the “transparencies of non-modal statements” proper to realism, like true and false ones. This means that both not-true and not-false positions do not only *not* exclude the corresponding terms along the vertical axis, but stand with them in a relation of implication, which, nonetheless, is in no way symmetrical: “denial implies negation while subtly differing from it, just as assertion implies affirmation without exactly being affirmation.” This, then, should rather be intended as a relation of articulation, where “articulations emerge from particular people speaking at specific times and places, with all of the species-specific processing and culturally-conditioned expectations that implies” (ibid.).

But the terms of the semiotic square are implicated along the diagonal axis too, revealing what Hayles calls “a common concern with the limits of representation” (ibid.). The “elusive negativity” expressed by the not-true position at the bottom left of the semiotic square is worth considering in detail. This, in fact, is the position that mostly escapes the either/or alternative of both realism and anti-realism, being a kind of negativity that is neither negative nor positive, and is thus inassimilable: let us think of the inappropriate/d other in Min-ha’s terms as Haraway (1992) explains it, where the inappropriate/d other is not the untouched, authentic other, but the other that is not “originally fixed by Difference” and that stands in a “critical, deconstructive relationality, in a diffracting rather than reflecting (ratio)nality” (ibid.: 299).

Elusive negativity is, for Hayles, precisely what designates the position *at* the cusp:

The diagonal connecting true and not-true reveals their common concern with the limits of representation. At the positive (“true”) end of the diagonal, the limits imply that we cannot speak the truth. At the negative (“not-true”) end, they paradoxically perform the positive function of gesturing toward that which cannot be spoken. Elusive negativity, precisely because of its doubly negative position, opens onto the flux that cannot be represented in itself (Hayles [1991] 1997).

The signification of the cusp is obviously always ambiguous, depending on the result of the encounter between physical and semiotic constraints that allude both to the reality of the world and the reality of language—the Harawaian material-semiotic field—without fully representing them. Such a position recognizes that what we can get to know are, at least, the boundaries of the cusp; it thus bypasses not only realism but also relativism. As Hayles explains at the end of her text (ibid.), commenting on the notion of partial perspective elaborated by Haraway, it is not that we only partially see the truth in things while remaining ignorant of its totality. It is, rather, that partiality is the whole that we see as the result of contextual and specific interactions with the “flux.” That is why she insists on what happens “at the dividing line,” in between the two sides (Hayles et al. 1995: 34). So,

If it is true that “reality is what we do not see when we see,” then it is also true that “our interaction with reality is what we see when we see.” That interaction has two, not one, components—what we bring to it, and what the unmediated flux brings to it. […] Omitting the zone of interaction cuts out the very connectedness to the world that for me is at the center of understanding scientific epistemology (ibid.).

## Inhabiting figurations

Constrained constructivism presupposes a language of metaphors: the difference that passes between metaphors and descriptions is, for Hayles, the same that passes between consistency and congruence. Haraway prefers speaking of *figurations* to name such “performative images that can be inhabited” (Haraway 1997: 11). Even though figurations always retain a visual aspect, which is not a secondary element in our “visually saturated technoscientific culture” (ibid.; Haraway 2000: 102–103), figures need not be literally representational or mimetic. They “involve at least some kind of displacement that can trouble identifications and certainties” (Haraway 1997: 11): they are neither complete nor static pictures of the world, but are representationally adequate insofar as they keep their performativity, with all its contradictions, alive.

Braidotti (2003), in her postmetaphysical feminist philosophy of difference, explains that this distinction between figurations and metaphors is intended to overcome the classical dichotomy of identity and alterity. From a Deleuzian perspective, the figural, based on difference and becoming, is opposed to the traditional aesthetic category of the figurative (or traditional representation) which, on the contrary, is based on identification and analogy between sign and object (Braidotti 2002: 78 ff.; 2003: 48; 2006: 170). According to Braidotti, figurations map the metamorphoses and hybridizations of subjectivities in technoculture. Actually, figurations do not stand outside the world they describe, but are living maps and transformative accounts never detached from their geopolitical and historical locations; they serve to “represent what the system had declared off-limits” without, in turn, attributing a separate status to it, as if the representation of differences were an end in itself (Braidotti 2006: 170). Figurations do not reify nor romanticize alterity, but “materially embody stages of metamorphosis of a subject position towards all that the phallogocentric system does not want it to become” (Braidotti 2002: 13).

Whereas metaphors generally presuppose two distinct tracks—that of signs and that of things—and work at reducing the unfamiliar to the familiar by linking two meaning systems, of which one is considered inert and stable, so as to reduce the one to the other—like the practice of mapping traditionally does (cf. Smith and Katz 1993)—figurations maintain a reciprocity between the two orders of meaning that shed light on another kind of space (and on different subject positions): one that is relational, active and unfixed. They stress transition, interconnectedness, interaction and border-crossing, as opposed to individuation and distinction (Braidotti 2002 Met: 70). As Smith and Katz contend, discussing the function of spatial metaphors in contemporary social theory, reconceived metaphors can work as an “Alice’s passage through the looking glass,” since they also “have the reciprocal effect of revealing the familiar as not necessarily so familiar” (Smith and Katz 1993: 91). Haraway’s figurations rework precisely the unfixity that co-implicates the two sides of Hayles’s analysis, transforming an exterior relation of correspondence into a relation of co-implication. They are of the utmost importance, then, for a project of technoscience intended as a travelogue of “distributed, heterogenous, linked sociotechnical circulations” (Haraway 1997: 12).

Haraway traces the origin of the meaning of the practice of figuration back to the semiotics of Western Christian realism, on the one hand, and to Aristotelian rhetoric on the other (Haraway 1997: 9 ff.; 2000: 141). In the history of Catholicism, the literal and the figurative continuously intersect, and figures are attributed to the power to contain the development of events, either of salvation or of damnation—something which Haraway also devises in the millenaristic tone of many discourses of technoscience. Aristotle highlights the spatial character of figures of discourse: in his philosophy, “a figure is geometrical and rhetorical; topics and tropes are both spatial concepts” (Haraway 1997: 11). This spatial aspect is visible in the strong link that Haraway’s figurations, in fact, maintain with location, although clearly locations cannot be made to coincide with abstract space, but rather, as Braidotti (2003) emphasizes, outline a cartography of spatial power relations and make sense of the different positionalities that these define. Figurations, moreover, also retain a temporal aspect that is by no means developmental, but assumes the modality of “condensation, fusion and implosion” which is contrary to the modalities of “development, fulfilment and containment proper of figural realism” (Haraway 1997: 12). It is precisely this implosion of boundaries between subject and object, or between the material and the semiotic, that puts borders in a constructive and transformative tension rather than using them as dividing lines. Figurations are thus tropoi, in that they, according to Greek etymology, do not simply figure, but “turn” what they figure (Haraway 2008: 159).^4^

It is once again Braidotti who, drawing on Haraway, shows how Harawaian figurations can be employed to develop a “politically charged practice of alternative representation:”

Feminist theories of “politics of location” (Rich [1984] 1987), or “situated knowledges” (Haraway 1991) […] stress the material basis of alternative forms of representation, as well as their transgressive and transformative potential. In feminism, these ideas are coupled with that of epistemological and political accountability (Harding 1987), that is the practice that consists in unveiling the power locations which one inevitably inhabits as the site of one’s identity (Braidotti 1999: 91–92).

This alternative practice, as Haraway repeats, can be delinked from the theologics of representation that revolves around reflection and reflexivity and their root in the mastery of light, which the tradition of feminist critique rightly dismisses, and be rather coupled with an optics that registers the passages of light rays through screens and slits, looking at the resonance and interference that light undergoes while passing through them.

## A different way of thinking about light

As a joke, albeit a serious one, Haraway affirms that semiotics is a science of four branches, “syntactics, semantics, pragmatics and diffraction” (Haraway 2000: 104). Intended as the production of difference patterns, diffraction, the fourth “optical” branch of semiotics, treats light differently from reflection, though, as we will see, not necessarily in opposition to representation. As Barad (2007) so poignantly summarizes,

First and foremost […] a diffractive methodology is a critical practice for making a difference in the world. It is a commitment to understanding which differences matter, how they matter, and for whom. It is a critical practice of engagement, not a distance-learning practice of reflecting from afar. (ibid.: 90)

Undoubtedly, reflection and reflexivity have their roots in representationalism (ibid.: 87), but the opposite is not necessarily true. I thus disagree with the reading that Campbell (2004: 174 ff.) offers of Haraway’s writings and their presumed evolution regarding the issue of representation, because I think that the model of articulation that a practice like diffraction presupposes is analogous to the way representations are reworked according to the notion of figuration, a project already pursued by Haraway in such writings as “Situated Knowledges.” I would not counterpose the latter to texts like “The Promises of Monsters” or “Modest Witness” where, according to Campbell, Haraway would abandon the representational model in favour of the diffractive one. Rather, what Haraway drops is the *metaphysics* of representations, while at the same time she articulates representations by means of diffractive practices, so as to render them still employable for feminist technoscience.

As we have seen, when Haraway retrieves a notion like that of location for her idea of situated knowledge, she is at the same time exposing, via Withehead, “the fallacy of misplaced concreteness” that lies at the core of either traditional realism or of traditional representationalism, both being based on an ontological distinction between representations and reality as well as on the existence of a distant and invisible representer (Haraway 1991; Barad 2007: 46 ff.). So, Barad’s belief in the dynamism and articulation of matter, which is not “a support, location, referent, or source of sustainability for discourse” or any other external force inscribing onto it, but “always already an ongoing historicity” (Barad 2003: 821), is not so different from Haraway’s faith in the historical embeddedness of figurations. It is worth repeating that Haraway never abandons representations nor opposes diffractions to them. If Barad thinks that we should leave representations behind decisively for “matters of practices/doings/actions” (ibid.: 802), Haraway is saying that seeing too is a doing and that we are responsible for the generativity of our visual practices (Haraway 1991). Accordingly, Barad, when discussing the functioning of scanning tunnelling microscopes (STM), which not only allow the visualization of but also the manipulation of atoms, notes that representations do not depict static objects out there, but are rather “condensations or traces of multiple practices of engagement” (Barad 2007: 53). Representations are performed as well as performing, so that we should rather talk about a set of *representational practices* that produce “what we take to be the evidence” (ibid.); our belief in them depends on historical and cultural variables, so that critically engaging with representations is always possible and, according to Haraway, also desirable (see also Barad 2007: 49). Only when they are critically engaged are metaphors put in motion, that is, activated through a process of translation, becoming effective, dynamic figurations rather than remaining reflective depictions of static givens.

When considering light, translation requires that we also consider that light has a history (Haraway 2000: 103). In fact, diffraction is a physical phenomenon that records the patterns of difference caused by the movements of rays resulting from the passage of light through a prism or a screen: “a diffraction pattern does not map where differences appear, but rather maps where the effects of difference appear” (Haraway 1992: 300). This process replaces the idea of a mimetic mirroring proper of reflection and refraction, or what Haraway calls the displacement “of the same elsewhere” (Haraway 1997: 273)—usually employed as a metaphor for the objectivity of science as well as for the traditional notion of artistic representation—in order to encompass interference, difference and interaction instead. “To make a difference in material-semiotic apparatuses,” says Haraway, we must be able “to diffract the rays of technoscience so that we get more promising interference patterns on the recording films of our lives and bodies” (ibid.: 16). The historicity of diffraction, then, lies in its situated, embodied character and in its being involved in facticity and in process making. This also entails a critique of the methodology of reflexivity and its infinite regression, which radical constructivism would counterpose to the realist option, since as we have already seen in Hayles’s critique of the separate domain of the observer, reflexivity too is trapped in a geometry of exclusions (the top line of Hayles’s semiotic square) whenever it poses difference as an absolutely unrelated alternative to sameness (Barad 2007: 72). “Reflexivity does not more than mirror mirroring” (ibid.: 88), because, even if the observers re-enter the picture, they still maintain a distance form the object of their gaze, foreclosing any “reading through” (ibid.: 90) the entanglements of phenomena and the production of borders.

Diffraction concerns the world of physical optics rather than that of geometrical optics. It describes the behaviour of waves when they encounter an obstacle, thus practically all optical phenomena; it also, contrary to geometrical optics, interrogates the nature of light. In physics, as Barad explains in her analysis, diffraction experiments are frequently used to compare the behaviour of waves to that of particles. One way to observe the phenomenon of diffraction, which the naked eye can easily notice when a pebble is launched into water or in the iridescence of a soap bubble, is the two-slit experiment, in which diffraction patterns resulting in bright or dark spots on a target screen—depending on the reciprocal enhancement or destruction of waves—are obtained when a light source passes, precisely, through a two-slit screen (ibid.: 71 ff.). According to classical physics, only waves can produce diffraction patterns, since only waves, not particles, can simultaneously occupy the same place. Barad, however, shows that quantum physics studies how particles can also behave like waves under certain circumstances. She then discusses the “modified” two-slit experiment at length, drawing on Niels Bohr’s diagrams; without entering into too much detail here, it suffices to say for the purpose of our argument that depending on the apparatus used in the two-slit experiment, that is, whether a “which path detector” is employed or not, matter, and light as well, are observed to manifest either particle or wave behaviour. This apparent paradox forces us to radically rethink the dualism that lies at the core of representationalism and the idea that “practices of representing have no effect on the object of investigation” (ibid.: 87), given that diffraction not only shows the entanglements of meaning and matter, but is itself an entangled phenomenon.

Thus, adopting a diffractive methodology, as Barad does drawing on Haraway’s lesson, implies a profound rethinking of Western ontology and epistemology (ibid.: 83) because it replaces the analogical methodology, which consists in relating two separate entities by way of an external observer, with a methodology that shows how “*practices of knowing are material engagements that participate in (re)configuring the world*” (ibid.: 91). Producing differences is what establishes connections rather than reinforcing distinctions: As Haraway writes, “diffraction patterns are about a heterogeneous history, not originals” (Haraway 2000: 101). A representation is not a sign that mirrors a separate external referent; it is rather a diffractive practice that reveals the coemergence and the co-implication of both meaning and matter. Agency is redefined as precisely “a matter of intra-acting,” from which the “agential realism” at the core of Barad’s philosophy is derived: since “intra-actions are *constraining* but not determinate,” (my italics) intra-acting neither belongs to a completely free subjectivity nor to a fully determined reality, but rather happens in a material-semiotic field where “particular possibilities for acting exist at every moment, and these changing possibilities entail a responsibility to intervene in the world’s becoming, to contest and rework what matters and what is excluded from mattering” (Barad 2003: 826–827). Talking about constraining intra-actions brings us back to the idea of consistency theorized by Hayles, according to which, as we have seen, constraints are what enable us to select among viable, that is, consistent, rather than congruent representations, shifting representations from what that we could see to the “interaction with reality [that] we see when we see” (see above).

This very much complicates the notion of vision as well as that of location (and the situatedness of the observer), since it dismantles the exteriority on which both have traditionally relied, and replaces it with specific forms of connectivity as well as accountability. Even if the observer comes back, he/she does not stand in a separate domain, but is connected in continuous feedback loops with his/her cognitive processes, since the closure of the observer’s domain is never pregiven, but always achieved (Hayles 1995: 78). Even as observers, we take part, writes Barad, in the “world’s differential becoming” (Barad 2007: 91) in which our knowledge enacts the world engaging in “specific worldly configurations” from the inside (ibid.).

## Conclusion

As Haraway notes, since we as humans need a “different *kind* of theory of mediations” (Haraway 2008: 174), new representational practices rather than new representations are required to make differences rather than merely see them. Since feminist theory has shown the criticality as well as the importance of a notion like that of representation, representations cannot be easily dismissed but should rather be reworked and signified according to alternative practices and wider semiotic frameworks. Adopting a performative idiom as a substitution for the representational one, thus getting completely rid of representations, leaves a series of questions unresolved, as Hayles and Haraway particularly highlight. These concern the domain of the observer as much as the status of what is observable, and most of all, that which relates the two sides, the sign and reality, or meaning and matter (Barad 2007).

The theory of constrained constructivism elaborated by Hayles ([1991] 1997) tries to formulate the viability of representations through the idea that they can never be congruent with reality but, rather, be consistent with it. Even if we do not get to know reality through representations, we can nonetheless “ride the cusp” that separates and at the same time connects us with the flux, touching the limit of representation (and, also, the limit of the knowability of reality). Modifying Greimas’s Square, Hayles proposes that we define the position at the cusp in terms of “elusive negativity,” a double negativity that connects us with the dividing line where we meet our interactions with reality and our representations of it as well.

This zone of intra-action is what Haraway’s practice of alternative representation goes through in order “to diffract the rays of technoscience” (Haraway 1997: 16). Haraway’s notions of figuration and of diffraction serve to displace fixed identities and put boundaries in constructive tension, requiring engagement rather than distancing. While Barad recognizes the importance of diffraction as a generative practice and interprets this notion in a non-representational way in her philosophy of agential realism, I have tried to argue that there is no need to oppose diffractions to representations, since what Haraway abandons is, first and foremost, the metaphysics of representation, but not the performativity of images which can be read through and used to *read through* at the same time.

We configure our world and establish connections with it through our ways of seeing. Diffraction, so intended, does not simply regard our visual field, but is a practice that invests our knowledge, our imaginary and our practices at the same time: it is, as Haraway writes, “a […] technology for making consequential meanings” (Haraway 1997: 273). Productive interruption, as well as reciprocal reinforcement, is allowed by diffractions and their unpredictable and unintended effects: different realities and unforeseen possibilities can emerge from diffractive practices (Haraway in Schneider 2005: 150).

## Footnotes

1 For a detailed discussion on the implications of a technoscientific approach see the articles contained in the special issue of Poiesis Prax (vol. 7, no. 1–2, 2010) entitled “Focus: Technoscience and Technology Assessment.”

2 If we, to take only one example, consider scholarship on visual studies, we observe that what is defined as the “pictorial turn,” an ambiguous concept in itself, is rooted in the acknowledgement of the non-mimetic, and in this sense non-representational, function of the image, which is now perceived as a “complex interplay” of relations rather than as the locus for the re-emergence of a pictorial presence (Mitchell 1994). Not so differently, the linguistic turn that philosophers such as Rorty (1967) advocated has actually been based on the same refusal of the model of representational transparency (and classical textuality) which governed traditional pictorialism. Visuality is so permeated with affect and desires that it is impossible to consider any visual representation independently from its effects, that is, the performative aspects that inhere in visuality, or what Thrift specifically calls the “effectivity” of the world (Thrift 2008: 113).

3 These notions of cusp and flux recall the concept of “double contingency” in Luhmann’s theory, which regulates the way Ego and Alter “intra-act,” relating to each other both through the indeterminacy of their own autoreferentality and the determinability of their own selections (cf. Baraldi et al. 1990: 75 ff.).

4 Similarly, Latour distinguishes between “intermediaries” and “mediators,” where only the latter transform what they transport rather than simply carry it (Latour 2005: 39).
